# Designing and Analyzing In-Place Motor Tasks in Virtual Reality With Goal Functions

**DOI:** 10.1109/TNSRE.2024.3439500

**Published:** 2024-08-16

**Authors:** Robert M. Carrera, Chenxi Tao, Sunil K. Agrawal

**Affiliations:** Department of Biomedical Engineering, Columbia University, New York, NY 10032 USA; Department of Mechanical Engineering, Georgia Institute of Technology, Atlanta, GA 30332 USA; Department of Mechanical Engineering, Columbia University, New York, NY 10032 USA

**Keywords:** Motor coordination, virtual reality, patient rehabilitation

## Abstract

Goal functions make virtual goal-oriented motor tasks easier to analyze and manipulate by explicitly linking movement to outcome. However, they have only been used to study constrained (e.g., planar) upper limb movements. We present a design framework for integrating goal functions with unconstrained postural and upper limb movements in a virtual reality (VR) device. VR tasks designed with the framework can mimic unconstrained natural motions and thus train a range of functional movements yet remain analytically tractable. We created three in-place VR motor tasks: a bow-and-arrow, a reach-and-strike, and a punching bag task. Each task was adjusted to subject-specific workspace limits and anthropometrics. We studied the effects of 3 days of practice and 3 reach/lean distances on task performance in 12 healthy adults. Subjects performed all tasks on day 1 with moderate proficiency and improved with practice at all reach/lean distances. Task-specific results showed that performance decreased and movement variability increased near the edge of the reaching workspace; viewing angles and the imperfect depth cues in VR likely led to biases in performance and practice could attenuate the former effect; in reach-and-strike, subjects learned movement patterns similar to those seen in a real-world striking sport. These results show that our framework can deliver tasks useful for analyzing and training motor performance and can guide future in-place motor training. Post-hoc, we demonstrated the feasibility of generalizable methods that adjust required movement speeds and task difficulty for impaired populations.

## I. Introduction

For people with a physical disability, deficits in postural and upper limb motor control can prevent stable standing and walking and negatively impact independence and educational and professional involvement [1].

Various movements have been used to train posture and upper limb motor control. Reaching tasks are common in both postural and upper limb rehabilitation [2]. Our lab has used activities including boxing a target and playing simplified “catch” with both hands to conduct postural training in children with CP [3]. Many studies exploring rehabilitative motor tasks in virtual environments (VEs) use commercially available exercise games focusing on sports, such as those on Microsoft Kinect or the Nintendo Wii [4], [5]. Some commercial platforms like IREX and BioRescue offer virtual tasks tailored for rehabilitation, including arm movements to reach or intercept targets or whole-body movements to navigate 2D virtual environments [6], [7]. Studies using head-mounted display (HMD)-based rehabilitative VEs have included tasks such as planar reaching and boxing [8]. However, whether delivered by a therapist or in a VE, subject movements in all but the simplest tasks are rarely analyzed. Instead, intermittent clinically validated assessments or simple measures like COP excursion or maximal reach distance are used to track functional recovery. Reviews of studies using IREX or custom HMD-based rehabilitation tasks show that only a few examined VE-based outcome measures, and these were limited to simple quantities like score, time to completion, or joint range of motion [8], [9].

Goal functions explicitly link subject movement to error in virtual motor tasks. A goal function $e=f(\vec{u},\;\vec{p})$ relates the scalar performance error, e, to the execution variables, $\vec{u}$ and the virtual environment parameters, $\vec{p}$ [10]. For example, in a dart-throwing task, the goal function incorporates the position of the target and the physics governing the flight of the dart after release in $\vec{p}$ and maps the selected execution variables $\vec{u}$ (e.g., dart position, velocity at release) to the radial error from the bullseye, e. The state of the virtual object just before execution, ${\vec{x}}_{e,1}$, may be incorporated in $\vec{p}$. Characteristics of motor skill acquisition include improvements in performance with practice, changes and eventual reduction in trial-to-trial variability (TTV), and the ability to retain the skill over time and adapt it to new contexts [11, pp. 257–259]. It is impossible to exactly reproduce a movement due to redundancy and sources of noise inherent to multiple levels of human physiology [12], [13]. As a result, changes in TTV while learning a new skill may represent exploration of different regions of the execution space or uncontrolled motor noise. Goal functions are ideal for tracking how with practice subjects learn to alter their movements, change TTV, and manage motor noise to eventually converge on a consistent motor strategy [14].

VE motor tasks with goal functions can provide clinically useful information. In a skittles task, subjects rotated a 1-DoF manipulandum with a release trigger to throw a tethered virtual ball to an on-screen target [14]. The goal function allowed for the definition of an optimal release time, revealing that Parkinson’s disease (PD) patients released the ball late, consistent with the movement initiation difficulties and bradykinesia typical of PD. This effect could be attenuated by using an unrelated release timing task as a warm-up [15]. However, previous VE-based motor tasks with goal functions have been implemented on custom-built 1- or 2-DoF input devices. These devices constrain the movement, often to a plane, and have not targeted 3D natural movements that could be used to train clinically relevant movement features such as maximal reaching or leaning, interlimb coordination, trunk-arm-hand steadiness, and movement rate control.

By implementing goal functions on commercial VR platforms that use unconstrained input devices, i.e., with 3 positional and 3 rotational DoF, one can design and analyze unconstrained, natural motor tasks that target movement features of clinical interest. However, using a 6-DoF input device requires that the task is performed in a reachable part of the reaching and postural workspace and is suited to the anthropometrics, movement speed, motor variability, and skill of the participant. The structure provided by a goal function gives the task designer control over the requirements for subject movement distances, positions, and speeds as well as the ability to influence overall task difficulty by manipulating the goal function parameters or via visual error modification.

These subject-specific task adjustments could be critical for supporting motor learning. Appropriate task difficulty is key: it places less demand on attentional resources and leads to a more subconscious search for a motor solution with better skill transference to stressful situations [16]; it may maximize interpretable information in the task to improve motor learning, according to the Challenge Point framework [17]; it is critical for subject engagement, enjoyment, and sense of competence and has been highlighted as a shortcoming of many current VE-based rehabilitative motor tasks [18], [19]. Along with task difficulty, the constraints-led theory of motor learning suggests that appropriate task scaling allows the subject to flexibly search for a motor solution suited to the constraints of their ability, the environment, and the task [20]. For instance, modifying features of a sport for children, such as court size and racket length in tennis or hoop height and ball size in basketball, has been shown to lead to more mature movement patterns, better performance, and faster motor learning [16]. Our approach can scale the task workspace and movement speed and target a moderate functional task difficulty while preserving the underlying goal function, which preserves the associated task constraints and perception-action couplings that are a critical part of learning a given task [16].

**Fig. 1.**
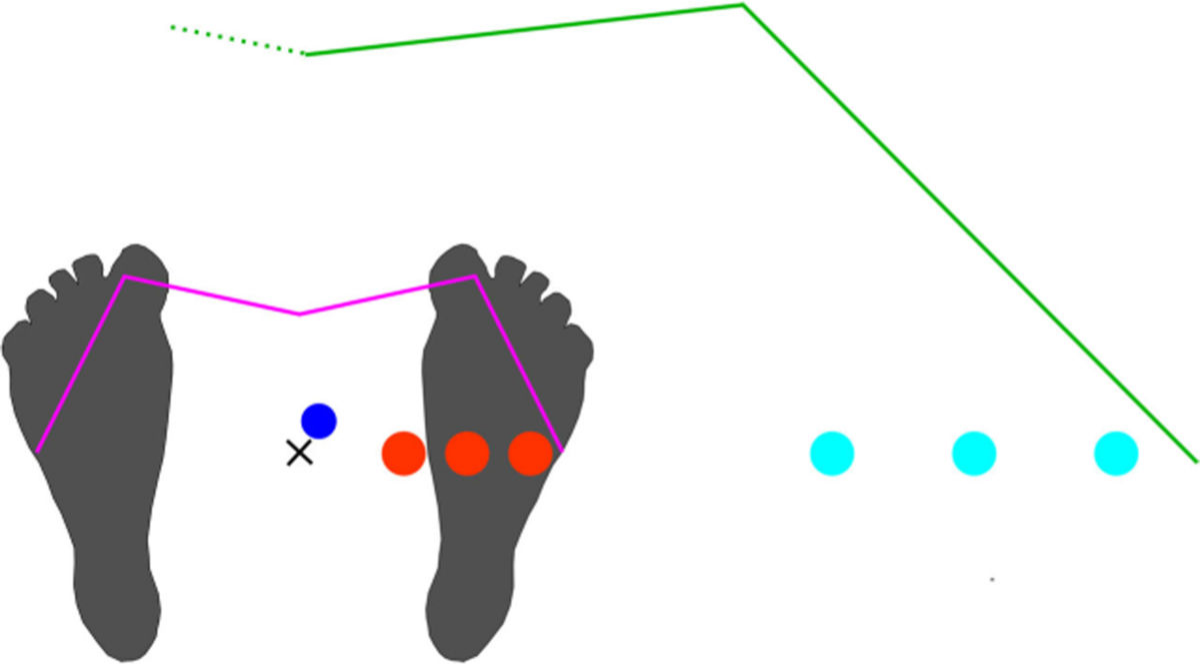
Using the lean/reach standing star test, the ground-plane postural workspace (pink line) of a control point (dark blue dot), such as the chest, can be established. Likewise, the ground-plane projection of the hand’s reaching workspace (green line) can be established. Postural targets (red circles) or reaching targets (light blue circles) can then be placed at varying percentages of the measured maximum postural excursion or reach distances.

We present a framework for designing unconstrained VE-based motor tasks with goal functions. We apply this framework to three novel in-place VR motor tasks: a bow-and-arrow task (BA), a reach-and-strike task (RS), and a punching bag task (PB). The three tasks highlight the flexibility of the framework and incorporate specific movement features targeted in rehabilitation. Each task required leaning (BA task) or reaching (RS, PB tasks) to 65% (“near”), 80% (“mid”), or 95% (“far”) of baseline postural/reaching limits, which were measured with the lean/reach standing star test (Fig. 1). We ask how lean/reach distance affected skilled task performance, with the hypothesis that increased lean or reach distance would increase postural effort and task difficulty and thus negatively affect task performance and learning rate. We analyze how a cohort of non-disabled subjects (n=12, 8 men and 4 women, ages 21 to 55 years, all right-handed) coordinated their posture, improved their performance over three days of practice, and were affected by reach/lean distance.

**Fig. 2.**
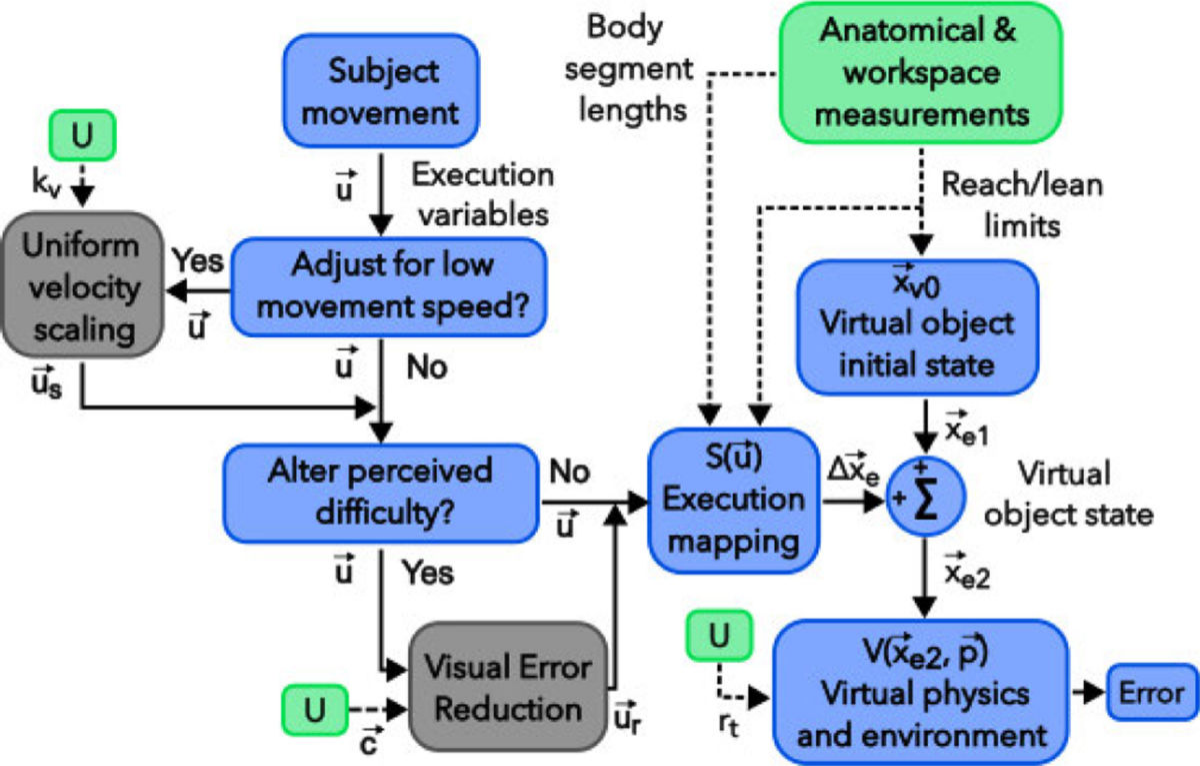
The goal function (blue boxes) maps from subject movement to a scalar error, e. $S(\vec{u})$ maps from the execution variables, $\vec{u}$, to a change in virtual object state, $\text{Δ}{\vec{x}}_{e}$. $V\left({\vec{x}}_{e2},\;\vec{p}\right)$ maps from the post-execution virtual object state ${\vec{x}}_{e2}$ to e and depends on parameters of the VE physics and layout in $\vec{p}$. Optionally, the task can include uniform velocity scaling to account for low movement speeds or target size adjustment and/or visual error modification to alter perceived difficulty. Setup for a task (green boxes) includes using anatomic and workspace measurements to place the virtual object within the postural or reaching workspace (position components of ${\vec{x}}_{V,0}$) and to normalize position- or distance-based execution variables passed to the execution mapping, $S(\vec{u})$. If velocity scaling, visual error modification, and/or target size adjustment are needed, the setup would also include using execution variable data from a prior block of trials, U, to determine the subject-specific velocity scaling constant, $k_{v}$, target radius, $r_{t}$, or visual error modification constants, $\vec{c}$.

We also conducted post-hoc validation of generalizable methods to adjust the tasks to different movement speed ranges and to tune task difficulty. Tasks are adapted to different movement speeds by uniformly scaling the velocity-based execution variables (Fig. 2). Perceived task difficulty can be altered by resizing the target or providing visual error modification. Our novel approach resizes the target radius, $r_{t}$, to observed TTV levels and applies visual error modification by altering execution-space deviations from the solution manifold (Fig. 2). Non-deterministic visual error reduction allowed children with spasticity to overcome their high motor variability and improve their movement strategy [21]. Our deterministic visual error modification generalizes the 1-DoF, task-specific visual error enhancement described by Hasson et al. and offers more control than non-deterministic methods [21], [22]. We also discuss selectively adjusting the error of certain execution variables, which could shift a subject’s attention to certain movement features and facilitate learning. For example, a shift of subject attention to certain task components improved skill acquisition in computer gaming [11, Ch. 18].

## II. Methods

### A. Framework for in-Place Task Design

To place virtual objects within the reaching/leaning workspace, we used upper limb length and distance limits from the lean/reach standing star test (Fig. 1). We frame the goal function as a two-step mapping from execution to performance (Fig. 2). The first step, which we call the execution mapping $S(\vec{u})$, maps from the execution variables, $\vec{u}$, and the pre-execution virtual object state, ${\vec{x}}_{e1}$, to the new state of the virtual object after execution, ${\vec{x}}_{e2}$, as

(1) $${\vec{x}}_{e2}={\vec{x}}_{e1}+S(\vec{u})$$

The execution mapping for each task is reported in Section II–C. The workspace-based parameters and/or anthropometrics can be incorporated into $S(\vec{u})$ to normalize position-or distance-based execution variables to the subject’s reachable workspace and proportions. For example, in the BA task, the string draw distance is normalized by upper limb length, and the lateral chest excursion is normalized by the baseline lateral chest excursion distance. The second step maps from ${\vec{x}}_{e2}$ to error and is determined by the VE physics, VE spatial arrangement, and the formula for error. This step is described by $V\left({\vec{x}}_{e2},\;\vec{p}\right)$ in Fig. 2. Section II–E below describes how subject-specific uniform velocity scaling, target resizing, and visual error modification could be used to modify a task for impaired groups.

### B. Procedure

Experimental protocols were conducted and participant consent was obtained under the Columbia University IRB, protocol AAAR6780, approved on 10/15/2022. On Day 1, subject height and upper limb segment lengths were measured. In all tasks, subjects stood with the outer edges of the feet at shoulder width. Subjects completed the lean/reach standing star task on Day 1, and these workspace limits were used in all subsequent tasks. See the supplemental video for task demonstrations.

Subjects practiced the BA, RS, and PB tasks on three consecutive days. Each task block contained an equal number of trials randomized at each lean or reach distance: near, mid, and far. Subjects completed 75 trials per day for the BA task (25 per lean distance), two blocks of 90 trials (30 per reach distance) per day for the RS task, and one block of 90 trials (30 per reach distance) per day for the PB task. For TNC-cost computations, task modification, and analysis, trials from each block were grouped by lean/reach distance.

Virtual tasks were programmed in Unity and administered with the Vive Pro VR system. Four base stations were set up around the subject to improve tracking performance, as multiple well-placed base stations can produce position errors on the order of 1mm or less [23]. All virtual tasks incorporated impact sounds such as hitting the bag, ball, or target played over the HMD’s speakers. Text feedback positioned near the target showed points earned in a trial (out of 100) and an encouraging message. Subjects wore a Vive tracker attached to the pelvis at the level of the iliac crest, the chest at a mid-sternal level, and the dorsal surface of each hand.

### C. Task Descriptions

**Fig. 3.**
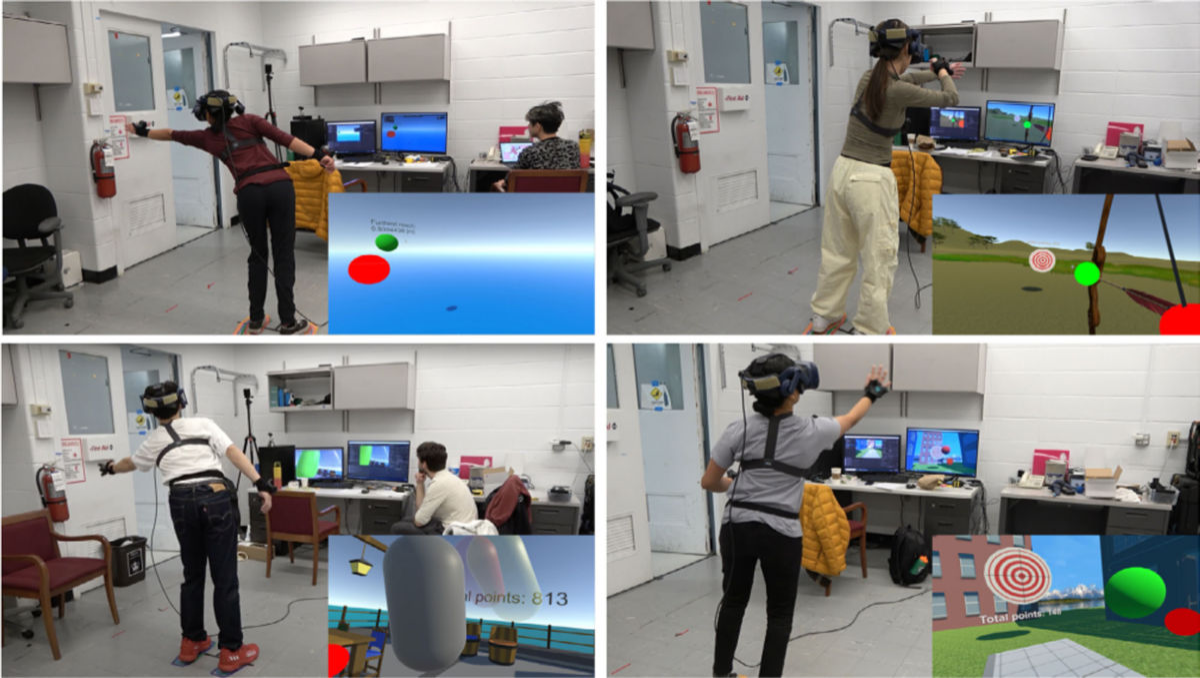
Clockwise from top left: the lean/reach standing star, bow-and-arrow, reach-and-strike, and punching bag tasks. Subject views of the task are shown as insets.

In the baseline lean/reach standing star test (Figs. 1, 3), subjects reached along 5 directions spaced at 45° intervals: right, forward-right, forward, forward-left, and left. Subjects were asked to maximally lean and reach for a floating ball with a vertical position at the neutral-stance height of the HMD.

The BA task was a sequenced task requiring leaning, bimanual coordination, and posture-arm-hand steadiness. The bow center was placed at 90% of the upper limb length forward from the subject and laterally on the side of the dominant arm at 65% (near-lean), 80% (mid-lean), and 95% (far-lean) of the baseline lateral chest limit. As the subject leaned laterally towards the dominant arm, the bow rotated about the vertical axis towards a static target on the non-dominant side. For a perfect shot, the subject had to hold the non-dominant hand near the bow grip while leaning to the correct chest excursion distance for the given condition, draw the bowstring by placing the dominant hand near the string and pulling “backwards” in the plane containing the bow grip and string to 65% of upper limb length, and then fire by moving the dominant hand away from this plane by more than 0.14 m. The subject had to keep their non-dominant hand within 0.08 m of the bow grip while drawing the bowstring or the arrow would release, no points would be assigned, and that trial was marked as a misfire. The two execution variables for the task were the lateral excursion of the chest normalized by baseline lateral excursion and the bowstring draw length normalized by upper limb length. The execution mapping relates the execution variables to changes in the arrow state (arrow position [x,y,z], velocity $\left[v_{x},\;v_{y},\;v_{z}\right]$) by

(2) $${\vec{x}}_{e2}={\vec{x}}_{e1}+S(\vec{u})=\left[\begin{array}{c} x_{1}\\ y_{1}\\ z_{1}\\ v_{x1}=0\\ v_{y1}=0\\ v_{z1}=0 \end{array}\right]+\left[\begin{array}{c} 0\\ 0\\ 0\\ kd_{pull}\sin\left(\theta_{bow}\right)\\ 0\\ kd_{pull}\cos\left(\theta_{bow}\right) \end{array}\right]$$

where the arrow position just before execution, ${\left[x_{1},\;y_{1},\;z_{1}\right]}^{T}$ was treated as the bow center regardless of pull distance, k is a scaling constant, $d_{pull}$ is the normalized string draw length, and $\theta_{bow}$ is the angle of the bow in the ground plane, set by the equation

(3) $$\theta_{bow}=\frac{x_{chest}}{x_{max}}\theta_{bow,max}$$

where $x_{chest}$ is the lateral excursion of the chest, $x_{max}$ is the baseline maximum lateral chest excursion, and $\theta_{bow,max}$ was set to 15°. The virtual physics governing the movement of the arrow were the equations for motion under gravity. The target was oriented such that it was perpendicular to the arrow path for $\theta_{bow}^{*}$, the optimal bow angle at release. Once initial arrow flight velocities were established, the time of flight to the target plane was

(4) $$t_{flight}=\frac{d_{target}}{{\|{\vec{v}}_{i}\|}_{2}\cos\left(\theta_{bow}^{*}-\theta_{bow}\right)}$$

where $d_{target}$ is the distance between the target and the bow origin and ${\vec{v}}_{i}$ is the initial arrow flight velocity vector. From flight time, the coordinates of the arrow when it reaches the target plane are computed, and the error is the radial distance from the bullseye to this point.

The RS task focused on gross arm movements and precise hand speed control. A green ball 0.31 m in diameter floated at the neutral-stance vertical position of the HMD, at 50% of the baseline forward reach distance, and at 65%, 80%, and 95% of the lateral baseline on the side of the dominant arm. Subjects were told to use their dominant hand to strike the ball to hit a target with a 2.0 m diameter and a bullseye located 4.0 meters in front of the ball and 1.0 m above the ball. The execution variables were the velocity components of the hand tracker, $\left[v_{xh},\;v_{yh},\;v_{zh}\right]$, at the moment of impact with the ball. As in the BA task, relevant ball states in ${\vec{x}}_{e1}$ and ${\vec{x}}_{e2}$ are ball position and velocity. The execution mapping related the execution variables to the change in the ball state,

(5) $${\vec{x}}_{e2}={\vec{x}}_{e1}+S(\vec{u})\;\;={\left[x_{1},\;y_{1},\;z_{1},\;v_{x1}=0,\;v_{y1}=0,\;v_{z1}=0\right]}^{T}\;\;+k{\left[0,\;0,\;0,\;v_{xh},\;v_{yh},\;v_{zh}\right]}^{T}$$

As in the BA task, the time of flight and coordinates at the moment of target plane intersection are computed from the initial ball flight velocity. Error is the distance from the bullseye to this point.

The PB task also focused on gross arm movements and precise hand speed control. A capsule-shaped punching bag was suspended from a pivot on the side of the subject’s non-dominant arm. The bag center was located at the neutral-stance vertical position of the HMD, at 50% of the baseline forward reach distance, at 65%, 80%, and 95% of the baseline lateral reach distance on the side of the non-dominant arm, and 1.118 m below its pivot point. The bag was 0.875 m long (bottom at y=2.875m, top at y=3.75m) and 0.438 m in diameter. The subject was asked to hit the bag such that the peak swing orientation matched the desired orientation, indicated by a translucent bag. The desired swing direction was 30 degrees towards the side of the dominant arm, which required reaching beyond the pivot to swing the bag medially. A red translucent bag briefly appeared after execution to indicate the peak swing orientation for that trial.

The execution variables were the ground plane velocity components of the hand tracker, $\left[v_{xh},\;v_{zh}\right]$, and the vertical distance from the bag pivot point to the point of impact, d. These determined the bag’s angular velocity, ω, and swing direction, ϕ. The execution mapping from execution variables to the change in bag state is described by

(6) $${\vec{x}}_{e2}={\vec{x}}_{e1}+S(\vec{u})=\left[\begin{array}{l} \omega_{1}=0\\ \phi_{1}=0 \end{array}\right]+\left[\begin{array}{c} kd\sqrt{v_{xh}^{2}+v_{zh}^{2}}\\ atan\left(\frac{v_{zh}}{v_{xh}}\right) \end{array}\right]$$

The dynamic equations for a point-mass pendulum were simplified using the small-angle assumption and yielded the closed-form solution

(7) $$\theta (t)=\theta_{max}cos(\omega t+\phi )$$

where $\theta_{max}$ was computed from the initial rotational kinetic energy. The axis-angle representation of the rotation from the desired to the observed peak swing orientation gave a scalar angular error.

### D. Computing TNC-Cost

The TNC-cost method characterizes how features of the distribution of a block of trials in execution space, denoted by the matrix U, affect performance. Let the rows of U represent individual trial executions, $\vec{u}$. T-cost evaluates how the position of the observations’ centroid in execution space affects performance, N-cost evaluates the effect of TTV on performance, and C-cost assesses how improvement in co-variation of the execution variables could affect performance [14]. When computing TNC-cost, possible optimized sets are created by manipulating the observed distribution of a block of trials in execution space. The optimum set, $U^{*}$, is the set with the lowest mean error. Each TNC-cost component is the mean error of the observed set minus that of the optimized set. We use TNC-cost to characterize task performance and in the visual error modification algorithm.

To compute T-cost, we created possible optimized sets by shifting the centroid of U to every point on a search grid without changing the distribution of the observations about the centroid. The search grid range and mesh size were chosen based on feasible limits. We used [start, step size, stop] search limits per execution variable for the BA task: normalized chest excursion ∈[0,0.0033,1.2], normalized draw distance ∈[0,0.01,1.5]; for the RS task: $v_{x}\in \;[-2.0,\;0.02,\;2.0]\;\text{m}/\text{s}$, $v_{y}\in \;[-1.0,\;0.1,\;7.0]\;\text{m}/\text{s}$, $v_{z}\in \;[-11.5,\;0.0656$, −1.0] m/s; for the PB task: $v_{x}\in \;[0,\;-0.0296,\;-5.0]\;\text{m}/\text{s}$, $v_{z}\in \;[0,\;-0.08,\;-8.0]\;\text{m}/\text{s}$, $d_{hit}\in \;[2.875,\;0.0056,\;3.458]\;\text{m}$.

To compute N-cost, possible optimized sets were computed by shortening the distance between all observed trial executions in U and the centroid of U in increments of 1%, without changing the centroid location. To examine the effect of the variance of each execution variable independently, we introduced an execution-variable-specific N-cost value, computed by shortening the distance between all observations in U and the centroid along only one execution space axis to generate possible optimized sets.

To compute C-cost for the PB and RS task, we extended a previously used greedy hill-climbing algorithm to the case of three execution variables [14]. In each batch of the algorithm, each value of the first execution variable was exhaustively paired with all values of the second and third execution variables by swapping values between observations. If the swaps are beneficial to the mean error of the set, they are accepted. The algorithm terminates when no swaps are accepted in a batch.

### E. Task Adjustments for Movement Speed and Difficulty

Beyond adjusting tasks to baseline reach/lean limits and anthropometrics, the tasks may need adjustment to account for cohort- or subject-specific movement speeds and abilities. We propose that modifying the execution variables in steps preceding the goal function is preferable to modifying the goal function itself. Tasks are designed such that a subject can easily view virtual objects of interest, the trial outcome, and any augmented visual feedback. Automatically changing the configuration of the VE would impact these viewing considerations. Preserving the goal function also means that non-disabled reference data for the unmanipulated task can provide a “typical” range for $\vec{u}$ and mean error, which can guide task adjustment parameter selection.

The task adjustment parameters are computed in three sequential steps: Step 1 determines a velocity scaling constant, $k_{v}$; Step 2 determines a new target radius, $r_{t}$; Step 3 produces a visual error modification vector, $\vec{c}$. Once the parameters have been computed, they can be applied to subsequent task performance as in Fig. 2. The parameter $k_{v}$ adjusts required movement speeds and $r_{t}$ and $\vec{c}$ modify the task difficulty.

A prior block of execution variable data from the subject, U, is needed to compute the parameters. Each of the n rows of U is a trial observation and the ith row is denoted ${\vec{u}}_{i}$. Here, we use a single prior block U to calculate $k_{v}$ in Step 1, use the scaled execution variables $u_{s}=k_{v}⊙\vec{u}$ to compute $r_{t}$ in Step 2, and use ${\vec{u}}_{s}$ and the new $r_{t}$ in Step 3. In practice, the steps could be sequentially layered and computed from three subsequent blocks of observations.

For Step 1, we denote the columns of U corresponding to position- and velocity-based execution variables as $U_{p}$ and $U_{v}$, respectively, and their ith rows as ${\vec{u}}_{p,i}$, ${\vec{u}}_{v,i}$. Element-wise multiplication of $k_{v}$ with $U_{v}$ produces scaled observations $U_{vs}$, as $U_{vs}=k_{v}⊙U_{v}$. The parameter $k_{v}$ is selected such that $U_{vs}$ has its centroid within a desired operating region in execution space. The operating region limits, ${\vec{v}}_{min}$, used in the constraint equation

(8) $$\sum_{i=1}^{n}\frac{\left(k_{v}⊙{\vec{u}}_{v,i}\right)}{n}\geq {\vec{v}}_{min}$$

could be based on non-disabled reference values for $U_{v}$. Once computed, $k_{v}$ alters the effective movement speed of subsequent trials. Specifically, $k_{v}$ produces scaled execution variables ${\vec{u}}_{s}$ by element-wise multiplication with observed velocity-based execution variables, as ${\vec{u}}_{s}=\left[{\vec{u}}_{p},\;k_{v}⊙{\vec{u}}_{v}\right]$.

In Step 2, the effective target size is adjusted to the TTV observed in U. The effect of centroid bias is first removed from U by applying the T-cost algorithm, giving the T-cost-optimized $U^{*}$ as defined in Section II–D. Calculating N-cost (N) for the T-cost adjusted $U^{*}$ then yields a measure of the effect of motor noise assuming the subject can learn to reduce performance bias with practice. A new target radius, $r_{t}$, is chosen such that

(9) $$\frac{N}{r_{t}}<e_{max}$$

where $e_{max}$ is a desired threshold for TTV-related error. The new target radius is used in subsequent trials.

In Step 3, the vector $\vec{d}=\vec{u}-{\vec{u}}^{*}$ is computed for each trial in U. It represents the “error” or “fluctuation” vector from the nearest point on the solution manifold to the observed execution [10]. This fluctuation vector is modified by element-wise multiplication with the visual error modification vector $\vec{c}$ and added back to the point on the solution manifold to produce a new set of execution variables, ${\vec{u}}_{r}$,

(10) $${\vec{u}}_{r}=T\left({\vec{u}}^{*},\;\vec{d}\right)={\vec{u}}^{*}+{\left[c_{1},\;c_{2},\;\ldots ,\;c_{m}\right]}^{T}⊙\vec{d}$$

(11) $$\frac{\bar{e_{m}}}{r_{t}}\leq e_{max}$$

where m is the number of execution variables in $\vec{u}$ and $\bar{e_{m}}$ is the mean error of U after visual error modification. Algorithmic selection of $\vec{c}$ (e.g., grid-search) can ensure that the constraint in (11) is met. Using this $\vec{c}$ and computing pertrial ${\vec{u}}^{*}$ in real time, (10) can be used to produce ${\vec{u}}_{r}$ from $\vec{u}$ for subsequent trials. The visual error modification of (10) can be applied to any task with a goal function.

However, in addition to just altering perceived difficulty, specific components of error can be selectively manipulated, possibly shifting subject attention to certain movement components. As an example of selective error-reduction, we let $\vec{c}=\left[c_{1},\;c_{2},\;1\right]$ and the order of $\vec{u}$ be $\left[v_{x},\;v_{y},\;v_{z}\right]$ in (10) for the RS task. We reduced only one of $\left(c_{1},\;c_{2}\right)$ to less than 1 and left the other equal to 1, independently reducing lateral $\left(c_{1}<1\right)$ or vertical $\left(c_{2}<1\right)$ error in the target plane. This could allow the subject to focus on coordinating either lateral or upward and forward strike velocity. Note, however, that if every component of $\vec{c}$ cannot be reduced, the constraint of (11) may not be met, as only errors related to the affected axes can be reduced.

Depending on the task, attention-shifting may require adjustment of (10) to target attention to desired task-specific features. For example, in the PB task, we reasoned that one may want to shift attention to hitting the bag with the right speed while reducing emphasis on hit direction. To do so, we reduced swing angle error while preserving the inertia-like properties of the bag, such that a hit of a certain velocity always makes the bag swing to the same height. Letting the execution variable elements have the order $\vec{u}=\left[v_{x},\;v_{y},\;d\right]$, we set $c_{1}=c_{2}<1$ and $c_{3}=1$ in order to scale the velocity-based components of $\vec{u}$. A new set of execution variables, ${\vec{u}}_{s}$, was computed that passed through a point $u_{s}$ with a reduced fluctuation vector, as in (10), but with a magnitude equal to the observed $\vec{u}$,

(12) $${\vec{u}}_{s}=T\left({\vec{u}}^{*},\;\vec{d}\right)=∥\vec{u}{∥}_{2}\frac{\left({\vec{u}}^{*}+{\left[c_{1},\;c_{2},\;c_{3}\right]}^{T}⊙\vec{d}\right)}{∥\left({\vec{u}}^{*}+{\left[c_{1},\;c_{2},\;c_{3}\right]}^{T}⊙\vec{d}\right){∥}_{2}}$$

This had the effect of preserving bag swing height but reducing variability and error in the bag swing direction.

Using each observed block as U, we computed $k_{v}$, $r_{t}$, and $\vec{c}$ for the RS task and $\vec{c}$ for the PB task. When computing $\vec{c}$, we used independent lateral/vertical and swing direction error reduction for the RS and PB tasks, respectively.

### F. Data Reduction and Statistical Analysis

In the BA and RS tasks, trials falling short of the target could have very high errors, so we removed trials with errors more than 15 times the target radius (trials/block BA task: median = 0, max = 2; RS task: median = 0, max = 3). Outlier trials in error (> 3 standard deviations from the block mean error) were then removed (trials/block BA task: median = 0, max = 2; RS task: median = 0, max = 1; PB task: median = 1, max = 2).

Outcome variables common to all tasks included performance (error), means and standard deviations of the execution variables, TNC-cost, and pelvis and chest lateral excursion normalized by baseline chest excursion. For target tasks, mean radial error (MRE) is the mean distance from the bullseye to observed hit positions and reflects accuracy (BA, RS tasks), BVE represents spread about the centroid of hits in the target plane and reflects consistency (BA task), and centroid bias is the distance from the bullseye to the centroid of hits in the target plane (BA task) [24].

RS task outcomes included an execution space trajectory analysis. For strikes that hit the target, we considered the period from 1 s before strike until strike and then selected the on-target movement time window, i.e., the uninterrupted period spent moving with execution variables mapping to errors less than the target radius. We also examined the average lateral (x-axis) movement speed in the uninterrupted period before execution spent moving with speeds greater than 20% of strike speed.

Using the nlme package in R, a linear mixed model (LMM) was implemented with each outcome variable as the dependent variable, lean/reach distance as a factor, block number as a covariate, and the interaction between lean/reach distance and block number as an additional factor. Two random effects per subject were added, one corresponding to the block 1 intercept and one corresponding to the slope associated with the block number. This full model was compared to a nested model without the random effects, but random effects improved the model fit, as assessed with a likelihood ratio test (the anova() function in nlme), in all cases. Approximate normality of the residuals was confirmed by inspecting a Q-Q plot, homoscedasticity of the residuals was seen across the range of the dependent variable, and the presence of leverage points on the overall, fixed, and random parameters of the model were examined. No leverage points were present in the data.

## III. Results

The day 1 reach-lean limits normalized by subject height (reported as mean ± standard deviation) by direction (R = right, FR = forward right, F = forward, FL = forward left, L = left) for the chest were R: 0.152 ± 0.027; FR: 0.128 ± 0.026; F: 0.081 ± 0.047; FL: 0.126 ± 0.022; L: 0.155 ± 0.022 and for the hand were R: 0.554 ± 0.028; FR: 0.500 ± 0.035; F: 0.453 ± 0.036; FL: 0.498 ± 0.030; L: 0.553 ± 0.031.

In Table I, the results from the LMM analysis of the BA, RS, and PB tasks are reported. The table includes t-statistics and associated p-values for comparisons of the non-reference levels of the lean/reach distance factor, mid-and far-lean/reach, to the reference (ref) level, near-lean/reach. Each dependent variable has a per-condition intercept value associated with block 1 and a slope reporting value change per block. Near-lean/reach is compared against an intercept and slope of 0. The means and standard errors of the mean (SEM) for the non-reference level (mid-, far-reach/lean) intercepts and slopes are reported as changes from the reference level (near-reach/lean) intercept and slope. Note that despite the two comparisons for the intercept and slope parameters (near- vs. mid- and near- vs. far-), this study is exploratory and hypothesis-generating so we did not apply a multiple comparisons correction such as Bonferroni to the reported p-values.

## IV. Discussion

### A. Bow-and-Arrow Task

**Fig. 4.**
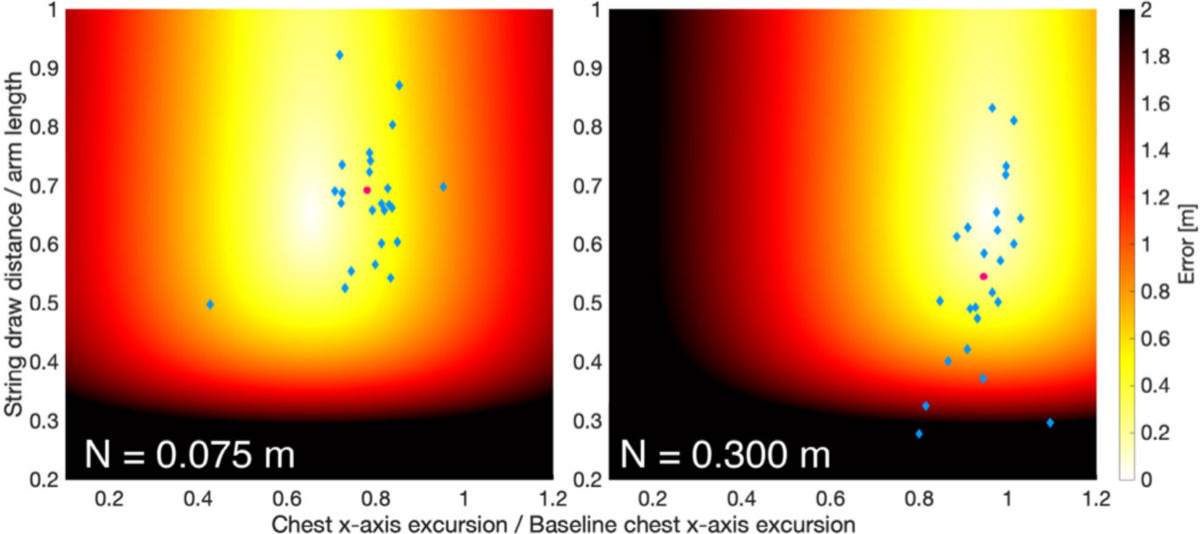
Representative distributions in execution space from subject 9, block 1 for the bow-and-arrow task (**left**: near-lean; **right**: far-lean). Heatmap colors correspond to arrow radial error associated with a point in execution space. Observed distributions (blue diamonds) and N-cost adjusted distributions (pink circles) are shown. Subjects tended to over-lean in near-lean and leaned close to optimally in far-lean in block 1, exemplified here. From near-lean to far-lean, N-cost (N) and $N_{dpull}$ increased for the cohort. Note that for subject 9, the N-cost optimization “shrank” the points to the centroid (scaling factor = 0).

As expected, greater lean distance elicited further pelvis $\left(d_{pelv}/d_{chest,b}\right)$ and chest $\left(d_{chest}/d_{chest,b}\right)$ excursion in block 1. Subjects were able to perform the BA task with a similar arrow MRE across lean distances in block 1. In far-lean, subjects showed significantly higher arrow BVE, N-cost, and $N_{dpull}$, lower normalized chest excursion error $\left(e_{d,chest}\right)$, and non-significantly trended towards a higher arrow centroid error (bias), higher arrow MRE, and more negative normalized pull distance error $\left(e_{d,pull}\right)$. In short, far lean distance increased the harmful effect of TTV, specifically TTV in the string draw distance, but it unexpectedly resulted in a more accurate chest excursion that led to a performance similar to near- and mid-lean distances in block 1. Increases in $N_{dpull}$ in far-lean may have been a result of the larger required chest excursion and more lateral target, which required a more lateral movement for drawing the bowstring and a more lateral gaze to view the target; these factors may have made consistently drawing the bowstring more difficult. The non-significant trend towards lower pull distances may have contributed to significant increases in $N_{dpull}$ by causing more “low” shots in the region where error rapidly increases (see Fig. 4).

The sighting phase in archery, in which the archer aligns a grip-mounted sight with the target while drawing the arrow back at a low constant velocity, is critical to archery performance [25]. However, because the bow was laterally displaced from the subject in this task, subjects may have had difficulty positioning and orienting the HMD to look down the line passing through the target and the bow center. This could have made it difficult to determine the optimal bow orientation and resulted in over-leaning in near- and mid-lean. As far-lean required chest excursions to 95% of baseline, over-leaning in far-lean was likely more difficult, explaining the more accurate chest positioning at far-lean in block 1.

In all lean conditions, $e_{d,chest}$ became more negative with practice, such that by block 3 subjects were on average leaning close to optimal in near-lean, moderately under-leaning in mid-lean, and substantially under-leaning in far-lean. Therefore, reductions in lean distance with practice improved performance in near-lean and worsened performance in far-lean. Declines in lean distance with practice are likely related to learning to lean to the optimal bow orientation without looking down the target-bow sight line by adapting motor commands to visual feedback of trial error [26]. This error correction process may have selectively adjusted for over-leaning error in near- and mid-lean target trials, leading to harmful motor corrections towards under-leaning in far-lean. Alternatively, decreasing motivation may have also contributed to reduced lean distance with practice.

Declining chest excursion standard deviation, $\sigma_{dchest}$, with practice surprisingly did not lead to a significant decrease in error for the cohort, as $N_{dchest}$ did not decline with practice. This highlights how metrics that incorporate the goal function, such as $N_{dchest}$, can directly explain how execution changes affect performance. This can help avoid drawing incorrect inferences from metrics that only consider the execution, such as $\sigma_{dchest}$. Performance improved in far-lean with practice despite increasing chest excursion error. This was likely caused in part by a trend (non-significant) towards lower pull distance error, supported by a trend towards declining $N_{dpull}$. However, no component of TNC-cost significantly decreased with practice at any lean distance, highlighting that different subjects improved through various means, including reductions in performance bias and in TTV.

### B. Reach-and-Strike Task

In block 1, greater reach distance elicited further chest excursion, whereas pelvis excursion only significantly increased at far-reach. Ball MRE was similar at near- and mid-reach but increased in far-reach. With practice, ball MRE decreased in all conditions and at a faster rate in the far-reach condition. As a possible explanation for faster learning in far-reach in the RS task, a higher error can lead to more opportunities for learning-relevant information to be gathered, as suggested by the Challenge Point framework. This was supported by results from a star-tracing task that showed higher initial errors correlated with faster learning but only in higher-difficulty tasks [27].

The TNC-cost analysis suggested that higher error at far-reach was due in part to higher T-cost, N-cost, and C-cost. Higher T-cost partially reflects a higher centroid bias in execution space. As the target was placed directly forward (along the negative z-axis) from the ball starting position, non-zero $v_{x}$ (lateral velocity) in this task is harmful to performance. Therefore, high $\left|v_{x}\right|$ in far-reach increased T-cost and error and its decline with practice at all lean distances contributed to reductions in T-cost and error. Increases in N-cost with larger reach distances indicate an increase in the negative effect of TTV with reach distance. Failure to decrease N-cost at near-reach distances suggests difficulty in decreasing harmful TTV beyond a certain level with the amount of practice provided. A higher C-cost in far-reach reflects poorer coordination of strike velocity components near the edge of the reaching workspace, although the effect was small relative to T- and N-cost increases. A decline in C-cost in far-reach with practice suggests coordination could be improved with training.

**Fig. 5.**
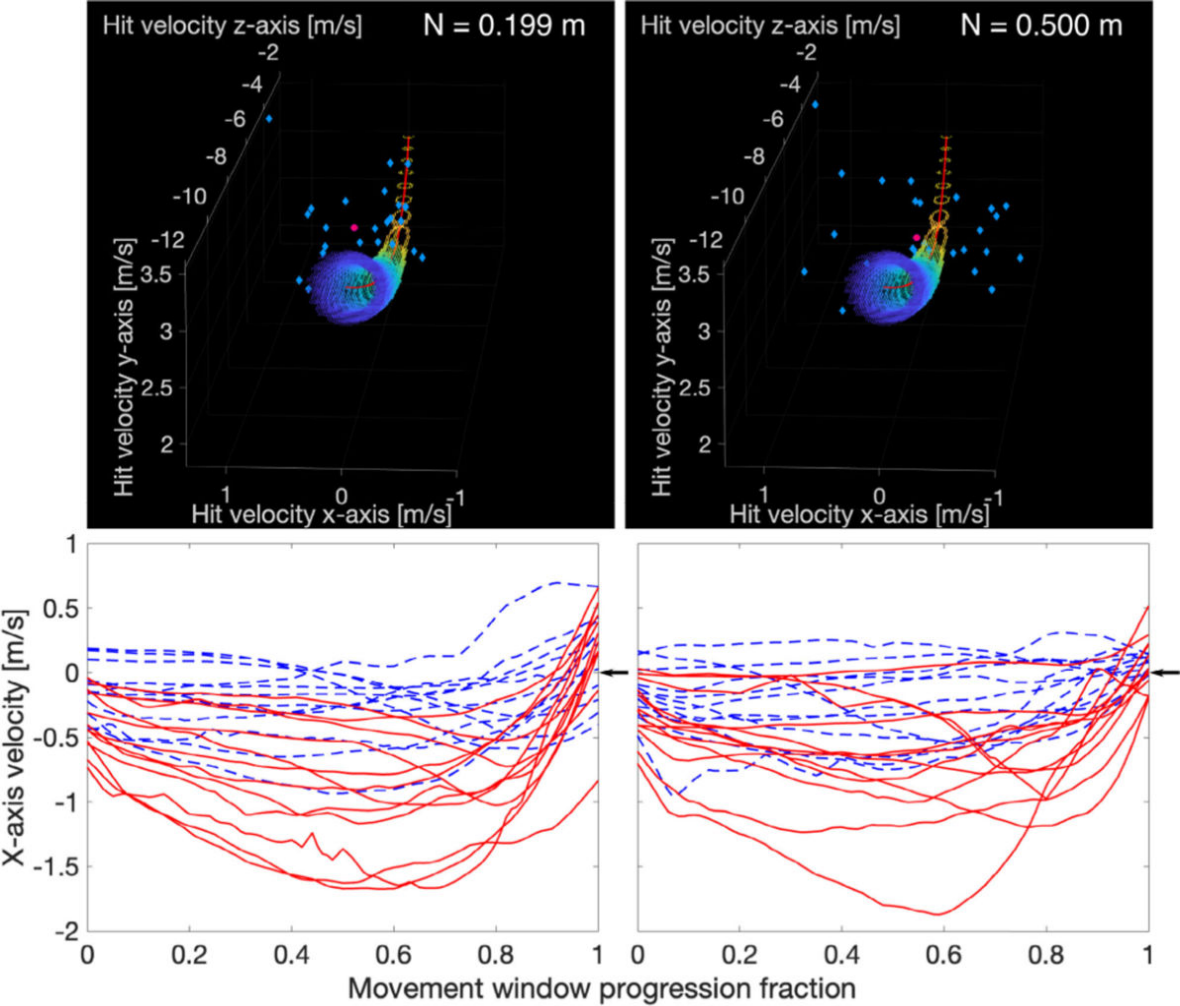
**Top row**: representative RS task distributions in execution space for near- (top left) and far-reach (top right) conditions for Subject 11, block 1. The solution manifold (red line) is shown along with points in the 8–12% target radius error region (small rainbow-colored dots). Observed distributions (blue diamonds) and N-cost optimized distributions (pink circles) are shown. N-cost, $N_{vx}$, and $v_{x}$-axis standard deviation increased substantially with reach distance for the cohort. For subject 11, the N-cost optimization “shrank” the points to the centroid (scaling factor = 0). **Bottom row**: mean x-axis (lateral) right-hand movement velocity histories in the pre-strike movement window are shown for each subject in near- (blue dashed lines) and far-reach (red lines) in block 1 (bottom left) and block 6 (bottom right). Negative $v_{x}$ signifies the hand is moving medially. With practice, subjects reduced mean strike $v_{x}$ in both near- and far-reach; note that from block 1 to block 6, $v_{x}$ at the moment of execution (movement window fraction = 1) was more tightly distributed around $v_{x}=0$ (black arrows) in near-reach and a tendency to strike with outward velocity was reduced in far-reach. In far-reach, subjects demonstrated a much higher mean $\left|v_{x}\right|$ in the pre-strike movement window in block 1, which significantly reduced with practice.

Execution-variable-specific N-cost components show how the TTV of individual execution variables contributed to N-cost. A large increase in the $v_{x}$-specific N-cost, $N_{vx}$, with reach distance contributed largely to increased error and N-cost (block 1 $N_{vx}$ values: intercept near (ref) = 0.079 ± 0.019; near vs null p < 0.001; intercept mid: 0.170 ± 0.025; mid vs. near p < 0.001; intercept far: 0.271 ± 0.025; far vs. near p < 0.001). Increases in $N_{vx}$ with reach distance likely resulted from large increases in $\sigma_{vx}$. The decline of $N_{vx}$ with practice in far-lean contributed largely to N-cost and error declines (Fig. 5; $N_{vx}$ slope values: near (ref) = 0.002 ± 0.005; near vs null p = 0.704; mid: −0.010 ± 0.007; mid vs. near p = 0.074; far: −0.022 ± 0.007; far vs. near p < 0.001). $N_{vy}$ also increased at far-reach in block 1 and declined with practice at far-reach (intercept near (ref) = 0.091 ± 0.013; near vs null p < 0.001; intercept mid: 0.099 ± 0.014; mid vs. near p = 0.585; intercept far: 0.124 ± 0.014; far vs. near p = 0.026; near slope (ref) = 0.005 ± 0.003; near vs null p = 0.127; mid slope: −0.001 ± 0.004; mid vs. near p = 0.078; far slope: −0.005 ± 0.004; far vs. near p = 0.007), as did $N_{vz}$ intercept and slope (intercept near (ref) = 0.005 ± 0.006; near vs null p = 0.445; intercept mid: 0.015 ± 0.008; mid vs. near p = 0.201; intercept far: 0.033 ± 0.008; far vs. near p < 0.001; slope near (ref) = 0.001 ± 0.002; near vs null p = 0.408; slope mid: −0.001 ± 0.002; mid vs. near p = 0.222; slope far: −0.004 ± 0.002; far vs. near p = 0.007), but these effects were of smaller magnitude.

Examination of the hand trajectory helps explain increases in $\sigma_{vx}$ and $N_{vx}$ with reach distance and decreases in mean $v_{x}\left(\mu_{vx}\right)$ and $N_{vx}$ with practice. Subjects tended to move their striking hand medially (negative $v_{x}$) throughout much of the pre-execution movement window at all reach distances (Fig. 5). However, medial velocities were higher with increasing reach distance, especially at far reach, as shown by increased mean $\left|v_{x}\right|$ magnitude in the pre-execution movement window $\left(\mu_{vx,tm}\right)$ (Fig. 5). Possibly, far-reach inward velocities were larger because the elbow was likely almost fully extended, meaning the arm is near an “elbow singularity”. With the hand near the target and the elbow fully extended, instantaneous movement of the hand along the hand-shoulder line becomes impossible, and forward movement of the hand is restricted to inwardly-arcing hand trajectories generated at the shoulder or, possibly, at the elbow [28, Ch. 5].

Subjects were able to moderately reduce $\mu_{vx,tm}$ with practice only in the far-reach condition. However, $v_{x}$ at the moment of execution was smaller than $\mu_{vx,tm}$, and $v_{x}$ at execution decreased with practice in all conditions. Subjects rapidly adjusted towards zero $v_{x}$ just before strike at all reach distances (Fig. 5). This suggests that subjects improved largely by timing their trajectories to have low $v_{x}$ at impact, instead of trying to produce trajectories with lower $v_{x}$ throughout the movement window (i.e., a low $\mu_{vx,tm}$). Subjects also spent more time moving with on-target velocities $\left(t_{ot,pre}\right)$ in near-reach than in far-reach, which may have reduced the importance of impact timing in near-reach. These data are consistent with observations from table tennis. After 1600 practice shots from the same location and with the same target, subjects reduced sagittal plane movement direction variability and maintained a more consistent bat travel direction for a brief period immediately preceding ball impact, likely to reduce the importance of impact timing, without reducing trajectory variability at earlier parts of the movement [29].

We computed task adjustment parameters post-hoc, with each block as $U,\;{\vec{v}}_{min}=\left[v_{x}=0,\;v_{y}=0,\;v_{z}=-11.0\right]\;\text{m}/\text{s}$ for velocity scaling, and $e_{max}=0.4\;\text{m}$ for target size adjustment and visual error reduction. The required $k_{v}$ almost doubled the effective hit velocity for most subjects ($k_{v}$ min: 1.0; 1st quartile: 1.814; median: 2.139; 3rd quartile: 2.53; max: 3.145) to achieve the high forward hit speed limit. For most blocks, N-cost normalized by target radius for the velocity-scaled, T-cost-adjusted observations was below $e_{max}$; for 37 blocks, typically modest target size increases were needed to meet this requirement ($r_{t}$ min: 1.002 m; 1st quartile: 1.040; median: 1.164m; 3rd quartile: 1.316 m; max: 2.621 m). For visual error reduction, 215 of the velocity-scaled blocks had mean errors normalized by target radius above $e_{max}$. For these blocks, $v_{x^{-}}\left(c_{1}<1\right)$ or $v_{y}$-axis $\left(c_{2}<1\right)$ error reduction successfully met this requirement 17.6 % and 68.5 % of the time, respectively. These results show that for the RS task, the amplification of TTV produced by a large $k_{v}$ could likely be compensated for with modest increases in target size, assuming the subject can learn to reduce bias with practice. Axis-specific visual error reduction would have to be chosen based on which axis manipulation could satisfy mean error requirements, or non-specific visual error reduction could be used.

### C. Punching Bag Task

Similar to the RS task, greater reach distance in block 1 elicited further chest excursion, but pelvis excursion only significantly increased at far-reach. At all reach distances, subjects swung the bag slightly too far laterally. This suggests that subjects had difficulty in precisely determining the desired swing direction or in responding to performance feedback. In general, the depth cues of HMD-based VEs lead to more natural movements than those of screen-based VEs but are poorer than real-world depth cues [30]. That swing direction error did not decrease with practice suggests that this was a persistent problem for many subjects. Subjects did, however, decrease total error and swing height error with practice at a similar rate at all reach distances.

**Fig. 6.**
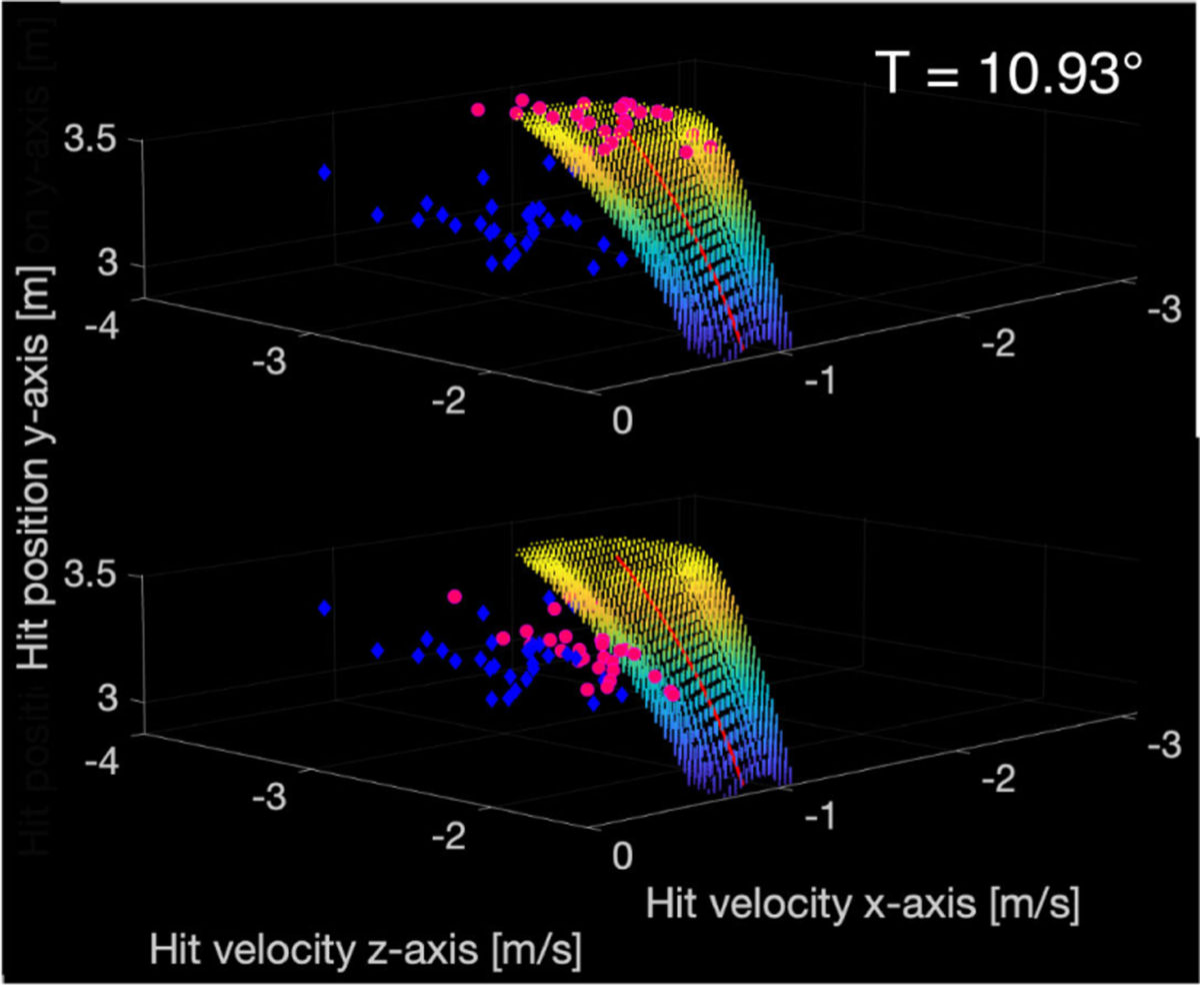
Representative distributions, from subject 10, block 1, far-reach, in execution space for the punching bag task. **Top**: The observed distribution (blue diamonds) and T-cost adjusted distribution (pink circles) are shown. T-cost and swing direction error increased in far-reach when compared to near-reach. Subjects likely had difficulty reaching far enough to direct the swing medially at the far-reach distance. **Bottom**: Post-hoc visual error reduction reduced swing angle error while preserving swing height. The observed distribution (blue diamonds) and adjusted distribution (pink circles) are shown.

At far-reach, subjects’ bag swing direction error increased to be more lateral, likely driving total mean error and T-cost increases (Fig. 6). Approaching the bag center along a heading towards the swing target required reaching an additional half of the bag radius beyond the bag center. That the cohort did not decrease bag swing direction error with practice at far reach suggests many subjects could not reach far enough. These results highlight how virtual object placement, size, and required angle of approach can affect performance.

Whereas increased swing direction error bias with reach distance may have masked increases in N-cost, taken together with the similar standard deviations of all execution variables at all reach distances, the impact of TTV on performance was likely similar at all reach distances. The need for higher movement speeds and to coordinate three velocity components in the RS task possibly led to increased movement variability and N-cost in far-reach observed for that task. C-cost declined with practice, indicating that improvements in execution variable coordination contributed to improvements in performance, likely reflecting better coordination of $v_{x}$ and $v_{z}$ to achieve reductions in swing height error.

For the 71 blocks with mean error $\bar{e}>e_{max}=10^{\circ }$, we computed visual error reduction constants $\vec{c}$ using the swing direction error reduction of (12). Swing angular error reduction was sufficient to reduce error below $e_{max}$ for 63 blocks. With moderate scaling coefficients ($c_{1}=c_{2}$ min: 0; 1st quartile: 0.351; median: 0.573; 3rd quartile: 0.799; max: 0.985), swing direction error was reduced (by min: 1.53%; 1st quartile: 20.0%; median: 43.7%; 3rd quartile: 64.6%; max: 100%), but swing height error was unchanged.

## V. Limitations and Conclusion

For in-place motor tasks, it is important to understand how training in different regions of the leaning/reaching workspace might impact postural involvement, performance, or motor learning. Normalizing the tasks to workspace limits and anthropometrics allowed subjects to immediately perform the tasks with adequate proficiency and to improve with practice at all lean/reach distances. Reaching or leaning further elicited further chest excursion and pelvic excursion. Our specific task analyses revealed that performance deteriorated in the far-lean/reach condition and suggested that the harmful impact of TTV (i.e., N-cost) increased at the edge of the reaching/postural workspace in the RS and BA tasks. In the BA task, the harmful impact of viewing angles on performance could be reduced with practice, which was likely dependent on visual feedback, while imperfect depth cues impairing the interpretation of feedback likely led to a subtle bias in swing angle in the PB task. Target size, position, and required angle of approach affected far-reach task performance in the PB task.

Contrary to our hypothesis, improvement occurred at similar rates at all lean/reach distances except in the RS task, where improvement was faster in far-reach. Prior work showing that adaptation to a novel force field transfers across the workspace and across different limb states suggests that learning of task dynamics at one lean/reach distance could transfer to task performance at other lean/reach distances, perhaps contributing to similar improvement rates in all conditions [31], [32]. Other factors may help the motor system distinguish and learn the required movements at each lean/reach condition. For example, both different movement locations and different movement follow-through can be important state-change cues that enable the motor system to form and recall distinct motor memories needed for task performance [32], [33]. These mechanisms could help in learning the presented tasks at the different lean/reach distances, as each workspace location required postural or limb state changes and likely led to changes in follow-through. The experimental control afforded by VR tasks designed with goal functions could be used in future experiments designed to probe how these and other factors influence motor learning in naturalistic movements.

We demonstrated methods for adjusting required movement speeds, target sizes, and visual error that, in theory, could enable a subject to immediately perform the adjusted task with moderate errors. As discussed, an appropriate and subject-specific task scaling and difficulty can lead to more enjoyment and engagement with the task and faster motor learning. However, reasonable target size increases may be limited, selection of a moderate target error, $e_{max}$, in (9) and (11) would likely have to be based on normative data and is task-specific, scaling applied to very low movement speeds could result in unmanageable amplification of TTV and subjects who cannot produce movements along directions needed for a task could not be accommodated.

We noted that selective visual error modification could shift attention to certain movement features. Visual error manipulation could also modify error to encourage motor learning or implicitly guide subjects to certain motor strategies [34]. Visual error amplification employed in the above-mentioned skittles task spurred subjects that had plateaued in performance to improve further [22]. Applying visual noise to regions of the execution space in a steering task promoted the adoption of specific motor strategies [35]. The framework presented here can provide error amplification, e.g., setting $\vec{c}$ components >1.0 in (10), or promote certain movement strategies, e.g., by applying visual error modification in selected execution space regions. More work must be done to determine how to best manipulate motor tasks to increase learning rate, enhance retention, encourage the adoption of robust motor strategies, and promote the transfer of learning to real-world tasks [34].

## Supplementary Material

supplemental material

## Figures and Tables

**TABLE I:** Linear Mixed Model Results by Task. Included Are t-Values (T) and p-Values (P) for the Significance of Model Terms, Model Coefficients (CF), and Their Standard Errors (SE). Row Names: Lean/Reach Distance Specifiers Near-, Mid-, and Far-, Slope (Lean/Reach Specifier x Block). Column Names: Mean Radial Error (MRE), T-Cost (T), N-Cost (N), C-Cost (C), $d_{pelv}/d_{chest,b}$ and $d_{chest}/d_{chest,b}$ Are Pelvis Excursion Distance and Chest Excursion Distance Normalized by Baseline Chest Excursion Distance (respectively), μ and σ Are the Mean and Standard Deviation of the Subscripted Quantity (respectively). In the Arrow Task Table, Centroid Radial Bias (Arrow Bias) and Bivariate Variable Error (BVE) Are Reported in the Target Frame. Centroid Bias Is Also Reported in Execution Space by Execution Variable $\left(u_{e,dchest},u_{e,dpull}\right)$. In The Reach-and-Strike Table, $t_{ot,pre}$ Is on-Target Time Pre-Execution. The Mean Lateral Velocity in the Pre-Execution Window Moving Over 20% of Strike Speed $\left(\mu_{v_{x},tm}\right)$ Is Reported. In the Punching Bag Table, Mean Total Error $\left(\mu_{e}\right)$, Error in Swing Direction $\left(\mu_{e,dir}\right)$, and Error in Swing Height $\left(\mu_{e,height}\right)$ Are Reported Separately. Significant p-Values (P <0.05) Are in Bold

Bow-and-arrow task													

	Arrow MRE [m]	Arrow bias [m]	Arrow BVE [m]	$\mu_{e,dchest}$	$\mu_{e,dpull}$	$\sigma_{dchest}$	$\sigma_{dpull}$	T[m]	N[m]	${\text{N}}_{dchest}\;[\text{m}]$	${\text{N}}_{dpull}\;[\text{m}]$	dpelv/dchest,b	dchest/dchest,b
Near- intercept t, p	10.04, <**0.001**	6.8, <**0.001**	7.89, <**0.001**	5.37, <**0.001**	2.67, **0.009**	8.47, <**0.001**	5.27, <**0.001**	4.29, <**0.001**	4.81, <**0.001**	4.44, <**0.001**	2.63, **0.010**	19.41, <**0.001**	49.81, <**0.001**
Near- intercept CF ± SE	0.344±0.034	0.229±0.034	0.292±0.037	0.076±0.014	0.104±0.039	0.074±0.009	0.14±0.026	0.121±0.028	0.109±0.023	0.043±0.010	0.056±0.021	0.489±0.025	0.73±0.015
Mid- intercept t, p	−0.07, 0.94	−0.5, 0.62	0.85, 0.4	−2.38, **0.02**	−0.28, 0.78	0.7, 0.48	1.11, 0.27	−0.54, 0.59	1.61, 0.11	1.60±0.112	1.57±0.119	3.60, <**0.001**	7.36, <**0.001**
Mid- intercept CF ± SE	−0.002±0.032	−0.02±0.039	0.027±0.032	−0.036±0.015	−0.006±0.022	0.004±0.006	0.018±0.016	−0.017±0.031	0.033±0.021	0.018±0.011	0.025±0.016	0.065±0.018	0.114±0.015
Far- intercept t, p	1.88, 0.06	1.55, 0.12	2.49, **0.01**	−5.19, <**0.001**	−1.6, 0.11	0.35, 0.72	0.56, 0.57	1.51, 0.14	2.2, **0.03**	1.23, 0.22	2.51, **0.014**	6.01, <**0.001**	14.04, <**0.001**
Far- intercept CF ± SE	0.061±0.032	0.061±0.039	0.079±0.032	−0.079±0.015	−0.036±0.022	0.002±0.006	0.009±0.016	0.046±0.031	0.045±0.021	0.014 ± 0.011	0.040 ± 0.016	0.109±0.018	0.217±0.015
(Near- x Block) t, p	−2.15, **0.03**	−1.06, 0.29	−2.12, **0.04**	−2.66, **0.009**	−0.35, 0.72	−2.04, **0.04**	−0.84, 0.4	−0.96, 0.34	−1.07, 0.29	−0.22, 0.82	−0.79, 0.43	−0.72, 0.47	−2.88, **0.005**
(Near- x Block) CF ± SE	−0.048±0.023	−0.029±0.028	−0.044±0.021	−0.025±0.01	−0.011±0.03	−0,009±0.004	−0.011±0.013	−0.02±0.021	−0.016±0.015	−0.001 ± 0.006	−0.010 ± 0.012	−0.013±0.018	−0.028±0.01
(Mid- x Block) t, p	0.25, 0.8	0.41, 0.68	−0.14, 0.89	−0.35, 0.73	0.35, 0.73	−0.56, 0.58	−0.37, 0.72	0.08, 0.94	−0.96, 0.34	−1.18, 0.240	−0.80, 0.42	−0.13, 0.9	−0.37, 0.71
(Mid- x Block) CF ± SE	0.006±0.025	0.013±0.031	−0.004±0.025	−0.004±0.012	0.006±0.017	−0.002±0.004	−0.005±0.012	0.002±0.024	−0.015±0.016	−0.010 ± 0.009	−0.010 ± 0.012	−0.002±0.014	−0.004±0.012
(Far- x Block) t, p	0.26, 0.79	0.27, 0.79	−1.23, 0.22	−0.8, 0.42	1.75, 0.08	−0.05, 0.96	0.46, 0.65	−0.26, 0.79	−1.97, 0.05	−1.31, 0.19	−1.73, 0.087	−0.01, 0.99	−0.65, 0.52
(Far- x Block) CF ± SE	0.007±0.025	0.008±0.031	−0.03±0.025	−0.009±0.012	0.03±0.017	0±0.004	0.006±0.012	−0.006±0.024	−0.031±0.016	−0.011 ± 0.009	−0.021 ± 0.012	0.00 ±0.014	−0.008±0.012

Reach-and-strike task														

	Ball MRE [m]	$\mu_{vx}\;[\text{m}/\text{s}]$	$\mu_{vy}\;[\text{m}/\text{s}]$	$\mu_{vz}\;[\text{m}/\text{s}]$	$\sigma_{vx}\;[\text{m}/\text{s}]$	$\sigma_{vy}\;[\text{m}/\text{s}]$	$\sigma_{vz}\;[\text{m}/\text{s}]$	T[m]	N[m]	C[m]	tot,pre[s]	$\mu_{vx,tm}\;[\text{m}/\text{s}]$.	dpelv/dchest,b	dchest/dchest,b
Near- intercept t, p	12.17, <**0.001**	7.42, <**0.001**	36.71, <**0.001**	−10.89, <**0.001**	3.74, <**0.001**	5.68, <**0.001**	4.35, <**0.001**	10.82, <**0.001**	5.1, <**0.001**	8.06, <**0.001**	13.82, <**0.001**	5.34, <**0.001**	3.4, <**0.001**	7.38, <**0.001**
Near- intercept CF ± SE	0.537±0.044	0.328±0.044	2.54±0.069	−5.418±0.497	0.474±0.127	0.668±0.118	1.277±0.294	0.337±0.031	0.177±0.035	0.101±0.013	0.052±0.004	0.428±0.08	0.265±0.078	0.444±0.06
Mid- intercept t, p	0.67, 0.5	−1.66, 0.1	−0.14, 0.89	−2.26, **0.02**	3.75, <**0.001**	2.26, **0.02**	2.95, **0.004**	−0.84, 0.4	4.61, <**0.001**	1.38, 0.17	−1.21, 0.23	3.13, **0.002**	1.75, 0.08	2.36, **0.02**
Mid- intercept CF ± SE	0.029±0.043	−0.09±0.055	−0.011 ±0.079	−0.474±0.209	0.333±0.089	0.315±0.139	0.977±0.331	−0.029±0.034	0.161±0.035	0.019±0.014	−0.004±0.003	0.176±0.056	0.073±0.042	0.098±0.042
Far- intercept t, p	6.55, <**0.001**	0.47, 0.64	−2.63, **0.009**	−1.9, 0.06	6.84, <**0.001**	1.4, 0.16	1.28, 0.2	3.85, <**0.001**	8.48, <**0.001**	3.93, <**0.001**	−4.8, <**0.001**	7.55, <**0.001**	3.69, <**0.001**	5.45, <**0.001**
Far- intercept CF ± SE	0.282±0.043	0.025±0.055	−0.207±0.079	−0.397±0.209	0.608±0.089	0.194±0.139	0.423±0.331	0.132±0.034	0.296±0.035	0.055±0.014	−0.016±0.003	0.425±0.056	0.154±0.042	0.227±0.042
(Near- x Block) t, p	−3.05, **0.003**	−4.48, <**0.001**	−2.53, **0.01**	2.86, **0.005**	−1.36, 0.17	−2.2, **0.03**	−1.84, 0.07	−2.58, **0.01**	1.27, 0.2	−1.27, 0.21	0.87, 0.39	−0.99, 0.32	−0.31, 0.76	−0.51, 0.61
(Near- x Block) CF ± SE	−0.03±0.01	−0.044±0.01	−0.036±0.014	0.19±0.066	−0.035±0.026	−0.056±0.025	−0.111±0.06	−0.016±0.006	0.011±0.009	−0.004±0.003	0.001±0.001	−0.015±0.015	−0.004±0.015	−0.005±0.01
(Mid- x Block) t, p	−0.07, 0.95	1.32, 0.19	0.19, 0.85	1.34, 0.18	−2.27, **0.02**	−1.73, 0.09	−1.98, 0.05	0.94, 0.35	−3.3, **0.001**	−1.46, 0.15	0.62, 0.53	−0.64, 0.52	−0.17, 0.86	0.33, 0.74
(Mid- x Block) CF ± SE	−0.001±0.011	0.018±0.014	0.004±0.02	0.072±0.054	−0.052±0.023	−0.062±0.036	−0.169±0.085	0.008±0.009	−0.03±0.009	−0.005±0.004	0.001±0.001	−0.009±0.014	−0.002±0.011	0.003±0.011
(Far- x Block) t, p	−2.5, **0.01**	0.9, 0.37	0.33, 0.74	0.37, 0.71	−3.37, <**0.001**	−0.29, 0.77	−0.43, 0.67	−1.08, 0.28	−4.9, <**0.001**	−2.37, **0.02**	1.85, 0.07	−2.1, **0.04**	−0.91, 0.37	−0.56, 0.58
(Far- x Block) CF ± SE	−0.028±0.011	0.013±0.014	0.007±0.02	0.02±0.054	−0.077±0.023	−0.01 ±0.036	−0.036±0.085	−0.01±0.009	−0.044±0.009	−0.009±0.004	0.002±0.001	−0.03±0.014	−0.01±0.011	−0.006±0.011

Punching bag task														

	$\mu_{e}\;[\text{deg}.]$	$\mu_{e,dir}\;[\text{deg}.]$	$\mu_{e,height}\;[\text{deg}.]$	$\mu_{vx}\;[\text{m}/\text{s}]$	$\mu_{vz}\;[\text{m}/\text{s}]$	$\mu_{d}\;[\text{m}]$	$\sigma_{vx}\;[\text{m}/\text{s}]$	$\sigma_{vz}\;[\text{m}/\text{s}]$	$\sigma_{d}\;[\text{m}]$	T[deg.]	N[deg.]	C[deg.]	dpelv/dchest,b	dchest/dchest,b
Near- intercept t, p	9.05, <**0.001**	3.6, <**0.001**	−2.72, **0.008**	−12.92, <**0.001**	−22.39, <**0.001**	114.42, <**0.001**	7.42, <**0.001**	9.35, <**0.001**	6.52, <**0.001**	4.68, <**0.001**	4.8, <**0.001**	7.58, <**0.001**	2.88, **0.005**	4.86, <**0.001**
Near- intercept CF ± SE	14,035±1.552	7.982±2.216	−4.216±1.549	−0.965±0.075	−2.365±0.106	3.106±0.027	0.382±0.051	0.561±0.06	0.051±0.008	6.758±1.444	5.515±1.15	3.091 ±0.408	0.187±0.065	0.293±0.06
Mid- intercept t, p	1.38, 0.17	1.87, 0.07	−0.41, 0.68	1.44, 0.15	−0.38, 0.7	−1.23, 0.22	−1.42, 0.16	−1.14, 0.26	−1.13, 0.26	2.84, **0.006**	−1.52, 0.13	−1.11, 0.27	1.93, 0.06	3.64, <**0.001**
Mid- intercept CF ± SE	1.862±1.349	3.065±1.642	−0.541±1.314	0.124±0.086	−0.029±0.075	−0.014±0.012	−0.081 ±0.057	−0.086±0.075	−0.007±0.006	3.027±1.067	−1.936±1.272	−0.418±0.377	0.084±0.044	0.188±0.052
Far- intercept t, p	3.05, **0.003**	3.01, **0.003**	−0.31, 0.76	2.36, **0.02**	−0.62, 0.54	−1.4, 0.16	−0.99, 0.33	−0.13, 0.89	−0.33, 0.74	4.35, <**0.001**	−0.85, 0.4	−0.03, 0.97	3.72, <**0.001**	7.42, <**0.001**
Far- intercept CF ± SE	4.115±1.349	4.941±1.642	−0.408±1.314	0.203±0.086	−0.047±0.075	−0.016±0.012	−0.056±0.057	−0.01±0.075	−0.002±0.006	4.642± 1.067	−1.076±1.272	−0.013±0.377	0.162±0.044	0.383±0.052
(Near- x Block) t, p	−3.04, **0.003**	−1.66, 0.1	3.00, **0.004**	−0.36, 0.72	2.91, **0.005**	−0.41, 0.68	−2.07, **0.04**	−4.18, <**0.001**	−0.92, 0.36	−1.78, 0.08	−1.31, 0.19	−2.74, **0.007**	0.14, 0.89	1.28, 0.2
(Near- x Block) CF ± SE	−1.708±0.561	−1.463±0.881	1.911±0.637	−0.01±0.029	0.125±0.043	−0.003±0.008	−0.04±0.019	−0.104±0.025	−0.004±0.004	−0.879±0.493	−0.565±0.432	−0.413±0.151	0.004±0.026	0.033±0.026
(Mid- x Block) t, p	−0.51, 0.61	−0.51, 0.61	−0.48, 0.63	−0.66, 0.51	−0.82, 0.42	0.82, 0.41	0.87, 0.38	1.32, 0.19	1.24, 0.22	−1.46, 0.15	1.03, 0.31	0.69, 0.49	0.86, 0.39	0.51, 0.61
(Mid- x Block) CF ± SE	−0.322±0.633	−0.389±0.77	−0.296±0.617	−0.027±0.04	−0.029±0.035	0.005±0.005	0.023±0.027	0.047±0.035	0.004±0.003	−0.732±0.501	0.615±0.597	0.123±0.177	0.018±0.02	0.012±0.024
(Far- x Block) t, p	−0.42, 0.68	−0.23, 0.82	−0.88, 0.38	−0.52, 0.6	−0.87, 0.39	0.11, 0.92	0.69, 0.49	0.88, 0.38	−0.35, 0.73	−0.91, 0.36	0.64, 0.52	−0.45, 0.65	0.51, 0.61	−0.37, 0.72
(Far- x Block) CF ± SE	−0.265±0.633	−0.177±0.77	−0.545±0.617	−0.021±0.04	−0.031±0.035	0.001±0.005	0.018±0.027	0.031±0.G3S	−0.001±0.003	−0.456±0.501	0.382±0.597	−0.08±0.177	0.01±0.02	−0.009±0.024
